# Dentistry in Court

**Published:** 1894-03

**Authors:** 


### Dentistry in Court.

For about two years and a half a case has been upon the records of the courts of Detroit in which C. H. Land, of Detroit, was plaintiff, and J. Taft, of Cincinnati, defendant. This is a case in which it was alleged by the plaintiff that the defendant had used to sundry persons language very derogatory and injurious to the reputation of the plaintiff.

This case was brought to trial on Friday, January 19, 1894. A large share of the evidence by the prosecution was given by the plaintiff, and consisted mainly in describing and explaining what he had accomplished in the way of new and improved appliances for use in dental practice. Evidence covering but a very few minutes was given by two witnesses who said they heard the defendant make the statements charged in this case.

The defendant had no knowledge of ever having made the statements as alleged in the charge. Prominent members of the profession of Detroit were called to the witness stand, and stated that the plaintiff had made many devices and alleged improvements in dentistry, but there seemed to be upon the part of each one a disapproval of some business methods practiced by Dr. Land, especially his methods of advertising, which they seemed to regard as altogether unprofessional.

After the evidence was completed the attorneys occupied from two to three hours in the discussion of the case before the jury; after which the jury went out for deliberation.

After some two hours or more they returned saying they could not agree; upon which they were dismissed by the court. After which it was learned that the jury stood as follows: Five for the plaintiff and seven for the defendant.

Thus the case rests at the present.

